# Bacterial community composition shifts in the gut of *Periplaneta americana* fed on different lignocellulosic materials

**DOI:** 10.1186/2193-1801-2-609

**Published:** 2013-11-15

**Authors:** Danielle Bertino-Grimaldi, Marcelo N Medeiros, Ricardo P Vieira, Alexander M Cardoso, Aline S Turque, Cynthia B Silveira, Rodolpho M Albano, Suzete Bressan-Nascimento, Elói S Garcia, Wanderley de Souza, Orlando B Martins, Ednildo A Machado

**Affiliations:** Laboratório de Entomologia Médica, Instituto de Biofísica Carlos Chagas Filho da Universidade Federal do Rio de Janeiro, Rio de Janeiro, Brasil; Diretoria de Metrologia Aplicada às Ciencias da Vida, Instituto Nacional de Metrologia Qualidade e Tecnologia, Rio de Janeiro, Brasil; Instituto de Bioquímica Médica, Universidade Federal do Rio de Janeiro, Rio de Janeiro, Brasil; Departamento de Bioquímica, Universidade do Estado do Rio de Janeiro, Rio de Janeiro, Brasil; Laboratório de Ultraestrutura Celular Hertha Meyer, Programa de Parasitologia e Biologia Celular, Instituto de Biofísica Carlos Chagas Filho, Universidade Federal do Rio de Janeiro (UFRJ), Rio de Janeiro, Brasil

**Keywords:** Sugarcane, Bagasse, Microbiota, 16S, Periplaneta

## Abstract

**Abstract:**

Cockroaches are insects that can accommodate diets of different composition, including lignocellulosic materials. Digestion of these compounds is achieved by the insect’s own enzymes and also by enzymes produced by gut symbionts. The presence of different and modular bacterial phyla on the cockroach gut tract suggests that this insect could be an interesting model to study the organization of gut bacterial communities associated with the digestion of different lignocellulosic diets. Thus, changes in the diversity of gut associated bacterial communities of insects exposed to such diets could give useful insights on how to improve hemicellulose and cellulose breakdown systems. In this work, through sequence analysis of 16S rRNA clone libraries, we compared the phylogenetic diversity and composition of gut associated bacteria in the cockroach *Periplaneta americana* collected in the wild-types or kept on two different diets: sugarcane bagasse and crystalline cellulose. These high fiber diets favor the predominance of some bacterial phyla, such as Firmicutes, when compared to wild-types cockroaches. Our data show a high bacterial diversity in *P. americana* gut, with communities composed mostly by the phyla Bacteroidetes, Firmicutes, Proteobacteria and Synergistetes. Our data show that the composition and diversity of gut bacterial communities could be modulated by diet composition. The increased presence of Firmicutes in sugarcane bagasse and crystalline cellulose-fed animals suggests that these bacteria are strongly involved in lignocellulose digestion in cockroach guts.

**Background:**

Cockroaches are omnivorous animals that can incorporate in their diets food of different composition, including lignocellulosic materials. Digestion of these compounds is achieved by the insect’s own enzymes and also by enzymes produced by gut symbiont. However, the influence of diet with different fiber contents on gut bacterial communities and how this affects the digestion of cockroaches is still unclear. The presence of some bacterial phyla on gut tract suggests that cockroaches could be an interesting model to study the organization of gut bacterial communities during digestion of different lignocellulosic diets. Knowledge about the changes in diversity of gut associated bacterial communities of insects exposed to such diets could give interesting insights on how to improve hemicellulose and cellulose breakdown systems.

**Methodology/principal findings:**

We compared the phylogenetic diversity and composition of gut associated bacteria in the cockroach *P. americana* caught on the wild or kept on two different diets: sugarcane bagasse and crystalline cellulose. For this purpose we constructed bacterial 16S rRNA gene libraries which showed that a diet rich in cellulose and sugarcane bagasse favors the predominance of some bacterial phyla, more remarkably *Firmicutes*, when compared to wild cockroaches. Rarefaction analysis, LIBSHUFF and UniFrac PCA comparisons showed that gene libraries of wild insects were the most diverse, followed by sugarcane bagasse fed and then cellulose fed animals. It is also noteworthy that cellulose and sugarcane bagasse gene libraries resemble each other.

**Conclusion/significance:**

Our data show a high bacterial diversity in *P. americana* gut, with communities composed mostly by the phyla *Bacteroidetes*, *Firmicutes*, *Proteobacteria* and *Synergistetes*. The composition and diversity of gut bacterial communities could be modulated by font of diet composition. The increased presence of *Firmicutes* in sugarcane bagasse and crystalline cellulose-fed animals suggests that these bacteria are strongly involved in lignocellulose digestion in cockroach guts.

## Introduction

Lignocellulosic materials, such as sugarcane bagasse, have been considered promising materials in the biofuel industry for the synthesis of second generation ethanol. However, the affordable production of these biofuels derived from plant biomass is currently dependent on the discovery of new enzymes to increase the efficiency of cellulose hydrolysis. In this context, animals such as insects which feed on plant material or wood may possess interesting biochemical pathways to promote an efficient hydrolysis of plant polymers, solving inherent problems to the digestion of lignocellulose, including detoxification of secondary plant phytochemicals and enzyme inhibitors (Morrison et al. 2009). In insects, the digestion of cellulose and hemicellulose has been observed in several orders such as Thysanura, Plecoptera, Orthoptera, Isoptera, Coleoptera, Trichoptera, Hymenoptera, Phasmida, Blattodea and Diptera (Sun and Scharf 2010).

Several cockroaches (including *P. americana*) are omnivorous insects that feed and survive on different food sources including cellulose-rich compounds. In this insect, the production of endogenous glucanases is predominantly associated to salivary glands and the midgut (Genta et al. 2003; Bignell 1981). The cockroaches, as well as low termites (Inward et al. 2007), digest these polymers by cooperation between two systems: endogenous insect enzymes and enzymes secreted by a variety of gut microorganisms, including protozoa and bacteria (Sun and Scharf 2010). The digestion of lignocellulosic compounds in insect guts, in a general view, comprises three basic stages involving insect and microbial enzymes: Stage I: hydrolysis, Stage II: oxidation and/or fermentation and Stage III acetogenesis and/or methanogenesis (including the participation of Archaea) (Hongo et al. 2005; Gill et al. 2006; Konig 2006; Hongoh 2010; Watanabe and Tokuda 2010). In insects that digest cellulosis, the trituration (grinding) of fiber material is arguably Stage I, for which both cockroaches and termites have a well-developed gizzard (Hongoh 2010). In termites, the anatomical adaptations of the gut form specialized micro-environmental chambers with different pH levels and redox potential that promote an important increase in concentrations and functionality of the intestinal enzymes. These chambers allow the effective breakdown of lignocellulosic biomass and a concomitant release of sugar monomers (Watanabe and Tokuda 2010). These microorganisms are absolutely essential to the digestion of many different animals, from insects to humans, and their diversity has been mainly studied by 16S rRNA sequence analysis. This approach has revealed the presence of several bacterial species that are mostly affiliated, but not restricted, to the phyla *Firmicutes*, *Actinobacteria*, *Bacterioidetes*, and *Spirochetes* (Hongo et al. 2005; Gill et al. 2006) that are strongly associated to the stage I of digestion.

Spatial characteristics of insect guts may harbor a significant population of specialized resident bacteria in these different microenvironments (Brune 1998; Dillon and Dillon 2004). Several other factors can influence microbiota composition of animal guts such as the host immune system, environmental microbial inputs, and the presence of specialized intestinal anatomical structures, the pH of distinct segments, the redox potential during food passage and also the diet. It has been shown that diet components alter gut microbiota composition in several organisms such as humans, pigs, dogs, snails and others (Leser et al. 2000; Konstantinov et al. 2002; Middelbos et al. 2010; Cardoso et al. 2012). Changing the foraging source from grain to hay in the diet, for example, can significantly change the bacterial population of bovine rumen (Tajima et al. 2001). Furthermore, in *P. americana*, a cellulose-rich diet induces a specific growth of the hindgut microbiota, mainly of methanogens and the anaerobic ciliate protozoan *Nyctotherus ovalis*, that could be involved in increasing cellulolytic activity and methane production (Gijzen and Barugahare 1992; Gijzen et al. 1994). Thus, the adaptation of the gut microbiota to dietary changes could be an important mechanism for digestion, especially for displaying new specific enzymes associated with the degradation of particular types of polymers.

Thus, the guts of termites and cockroaches may represent a large source of untapped microbial diversity and, in this sense, culture-independent methods provide a powerful opportunity to study the species richness of insect gut bacteria involved in plant cell wall deconstruction and possibly help to discover new microbial enzymes for biofuel production (Warnecke et al. 2007). Considering that there is little information on how diet affects the composition of gut microbiota in cockroaches, this work describes for the first time the influence of sugarcane bagasse and crystalline cellulose diets on *P. Americana* gut bacterial communities.

## Materials and methods

### Experimental design

**Figure 1:**
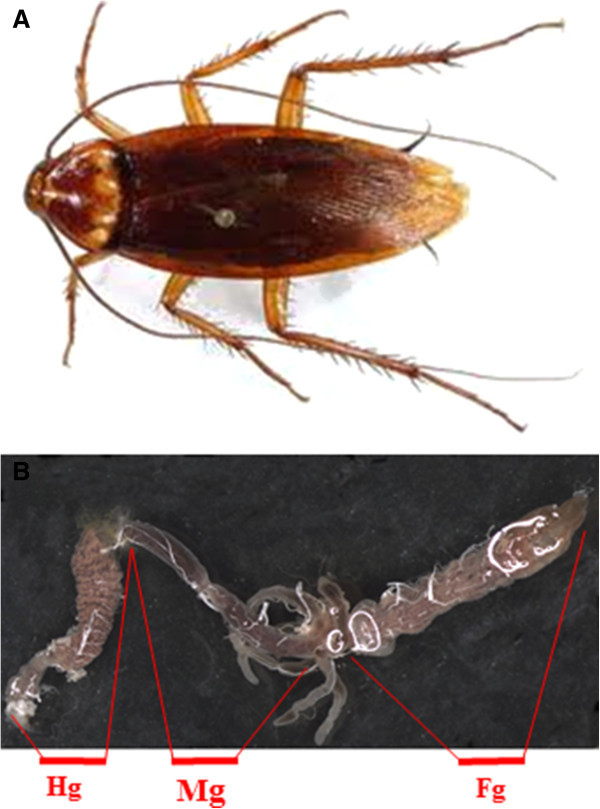
**Digestive system of** ***P. americana.*** **(A)** Adult insect and **(B)** Whole gut. FG–Foregut; MG–Midgut; HG; Hindgut.

Adult male and female cockroaches (Figure 1A) were selected from an established colony and kept under a natural light regime and fed with different diets. The animals were separated into individual containers and exclusively fed with dried finely mowed sugarcane bagasse or cellulose (Avicel® PH 101 Sigma Aldrish code product 11365, PA, USA) *ad libitum* for at least two weeks.

The industrial sugarcane bagasse used in this study has around 42% cellulose, 27% hemicellulose, 20% lignin, ashes (2%) and other substances (9%). All animals used in the experiments were able to consume the food provided and survived at least one month in these conditions. Water was offered in wet pieces of glass wool. Adult cockroaches (males and females) from wild-types were collected from sewage drains at Universidade Federal do Rio de Janeiro and immediately used.

### DNA extraction

Ten cockroaches of each of the three groups (wild-types, bagasse or cellulose) were dissected and had their gut contents (Figure 1B) removed in sterile PBS (phosphate-buffered saline 137 mM NaCl, 2.7 mM KCl, 10 mM Na_2_HPO_4,_ 2 mM KH_2_PO_4_ pH 7.4 10 mM) and pooled. Animals that, eventually, showed no gut content on dissection were discarded. Gut content was homogenized and centrifuged at 14,000 g for 10 minutes at 4°C. Supernatants were discarded and pellets were stored at-70°C until DNA extraction. Nucleic acids were isolated from the pellets by cell lysis with proteinase K and SDS, followed by phenol-chloroform extraction (Vieira et al. 2007). DNA integrity was checked on a 1% (w/v) agarose gel.

### Bacterial 16S rRNA gene library construction

PCR was performed in 50 μl reaction mixtures (2.5 mM MgCl_2_, 0.2 mM deoxynucleoside triphosphates, 50 ng of each primer, 0.25 U of High Fidelity Taq DNA polymerase (Promega), PCR buffer and 200 ng of DNA sample, using the universal bacterial primers 27BF (59-AGAGTTTGATCCTGGCTCAG-39) (Lane 1991) and 907RAB (59-TTTGAGTTT MCTTAACTGCC-39) 23-(Weisburg et al. 1991). PCR amplification was performed with a 5 min denaturing step at 94°C followed by 45 cycles of 94°C for 90 seconds, 55°C for 90 seconds, and 72°C for 2 min. The final cycle was an extension at 72°C for 10 min. PCR products were concentrated and purified with a GFx PCR DNA and Gel Band Purification Kit (GE Healthcare) after electrophoresis on a 1% (w/v) agarose gel. PCR products were cloned into the pGEM-T cloning vector (Promega) and used to transform competent *E. coli* DH10B cells. Positive colonies in the blue-white screen used for this vector were picked and frozen at -70°C.

### Sequence analyses and taxa identification

Approximately 96 clones from each of the three libraries were subjected to sequence analysis. Plasmid DNA from each clone (400 ng) was prepared and PCR sequencing reactions with primer 27BF were carried out using the DYEnamic ET terminator cycle-sequencing kit (GE Healthcare). Partial 16S rRNA sequences were obtained by capillary electrophoresis on a MegaBace1000 DNA analysis system (GE Healthcare). Chromatograms were transformed into Fasta format with Phred software (Edwing et al. 1998) and sequences with less than 300 bp and chimeras were removed prior to further analysis using MOTHUR (Schloss et al. 2009). A total of 216 (75/Bagasse; 79/cellulose; 62/Wild-types) valid sequences were compared with sequences in the Ribosomal Database Project II 26-(Cole et al. 2003). The Sequences were also analyzed by BLAST 27-(Altschul et al. 1990) searches in the GenBank database (http://www.ncbi.nlm.nih.gov) and were aligned with representative bacterial sequences obtained from the public databases using ClustalX software (Thompson et al. 1997). The partial 16S rRNA gene sequences generated in this study has been deposited in GenBank under accession numbers file barataseqin2: JX887210-JX887422.

### Biodiversity and phylogenetic analyses

Resampling and adjustment of the total number of sequence reads to identical sequencing depth was done before analysis (Gilbert et al. 2010). Sequences were clustered as OTUs at an overlap identity cutoff of 97% or 80% by MOTHUR software (Schloss et al. 2009). The diversity of OTUs and community overlaps were also examined using rarefaction analysis and Venn diagrams. The rarefaction analysis related the number of OTUs discovered to the number of samples taken, to discover whether additional sequences would discover additional taxa. The identity of each 16S rRNA sequence was determined by BLAST-n searches against the NCBI GenBank database. Phylogenetic trees were constructed for *P. americana* gut bacterial libraries with reference sequences from GenBank by the neighbor-joining algorithm based on distances calculated by the Kimura-2 method. This analysis was performed with the MEGA4 program (Kumar et al. 2001) and bootstrap analysis with 1000 replications was used. Tree topology and distribution of hits along the tree were uploaded to the UniFrac computational platform (Lozupone et al. 2007). UniFrac is a beta diversity metric analysis that quantifies community similarities based on phylogenetic relatedness. In order to visualize distribution patterns of bacterial communities we used the UniFrac metric to perform PCA highlighted by significance.

### Statistical comparison between 16S rRNA libraries

In an attempt to determine the differences between clone libraries, we applied LIBSHUFF statistics (Schloss et al. 2004) that uses Monte Carlo methods to generate homologous and heterologous coverage curves. Sequences were randomly shuffled 999 times between samples prior to the distance between curves being calculated using Cramer-von Mise statistic test. The DNADIST program of the PHYLIP package, using the Jukes-Cantor model for nucleotide substitution was used to generate the distance matrix analyzed by LIBSHUFF.

## Results

**Figure 2:**
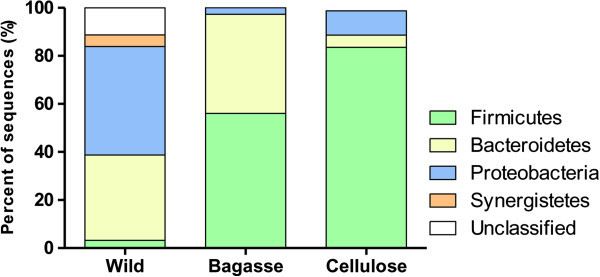
**Distribution bacterial phyla in 16S rRNA gene sequences from cockroaches submitted to different diets.** Gut bacterial DNA was obtained from cockroaches fed on sugarcane bagasse or cellulose (both for at least one week) and from wild-types cockroaches (collected from sewage). Clone libraries from amplified 16S rRNA genes were prepared and sequenced. Nucleotide sequences were submitted to the RDP Classifier tool at 80% bootstrap cutoffs.

A total of 216 valid sequences of approximately 700-800 bp were obtained from three 16S rRNA gene libraries, 62 from wild-types insects and 75 and 79 from sugarcane and cellulose fed cockroaches, respectively. The sequences were assigned to distinct taxonomic phyla with the RDP classifier tool (http://rdp.cme.msu.edu/classifierr/classifier.jsp). In wild-type animals, the *Proteobacteria* and *Bacteroidetes* were the predominant sequences of these phyla. Most sequences from the sugarcane and cellulose fed libraries were represented by the *Firmicutes* and *Bacteroidetes* phyla, as observed in the microbiota of other animals submitted to high fiber content diets (Ley et al. 2008). In the gut of cellulose fed animals, *Firmicutes* was by far the most abundant phylum (Figure 2), comprising more than 80% of the sequences. In wild-types animals, BLAST searches (http://blast.ncbi.nlm.nih.gov/Blast) performed with the bacterial sequences that could not be classified with the RDP tool (described as unclassified in Figure 2) retrieved only low similarity sequences suggesting that these microorganisms possibly represent new bacterial groups.

**Figure 3:**
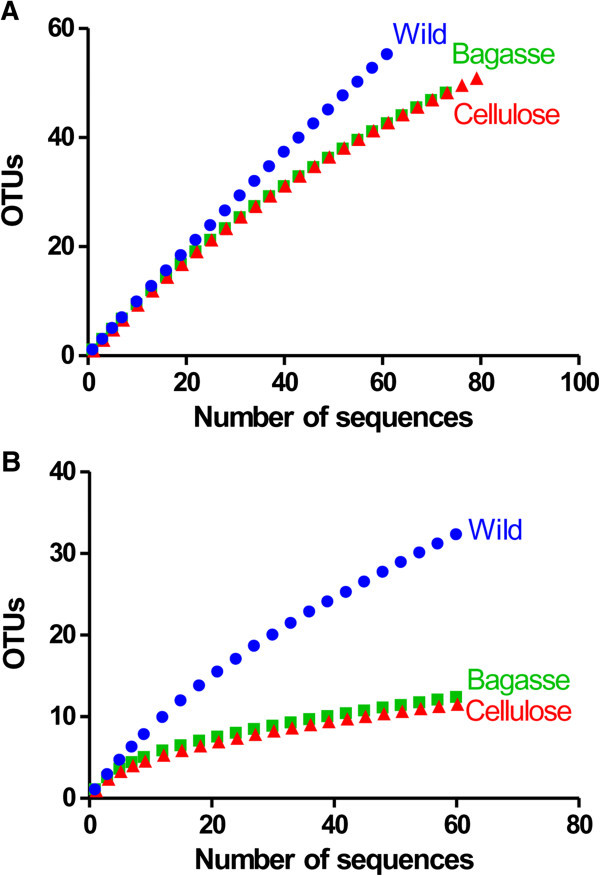
**Rarefaction analysis of 16S rRNA gene sequences from gut contents of wild-types cockroaches and those fed on sugarcane bagasse or cellulose.** The total number of sequences was plotted against unique OTUs defined by using a distance level of 97% **(A)** or 80% **(B)** using the furthest neighbor assignment algorithm in MOTHUR.

Rarefaction analysis indicates that the libraries exhibit different levels of diversity. Insects fed cellulose and sugarcane bagasse showed lower bacterial diversity than wild-types cockroaches. Rarefaction curves clustered at 97% (Figure 3A) or 80% (Figure 3B) similarity showed higher bacterial species diversity within the wild-types insects when compared with the insects fed cellulose-rich diets. The decline in the rate of OTUs detection at 80% cut-off denotes that only the most predominant sequences of these bacterial phyla have been observed in the animals fed higher fiber diets. Furthermore, at 97% the cut-off curves show that diversity at genus/species level was not entirely detected, mainly for wild-types animals. The number of clones sequenced from wild-types insects was not enough to cover the whole bacterial diversity, although diversity has been achieved almost completely in sugarcane bagasse or cellulose fed animals. Table 1 shows a comparison by LIBSHUFF statistics showing that bacterial community composition differed significantly between wild-types animals and higher fiber diet-fed (cellulose and sugarcane bagasse groups) cockroaches (p < 0.0001). On the other hand, when the libraries of insects fed a fiber-rich diet were compared between themselves, no significant differences were observed (p = 0.9355 and p = 0.0030).

**Table 1:** **Selected LIBSHUFF comparisons**

Comparison	dC_XY_Score	Significance
Wild-types x Bagasse	0.17079286	<0.0001
Bagasse x Wild-types	0.05764750	<0.0001
Wild-types x Cellulose	0.01280417	<0.0001
Cellulose x Wild-types	0.13229231	<0.0001
Cellulose x Bagasse	0.00228431	0.0030
Bagasse x Cellulose	0.00023490	0.9355

**Figure 4:**
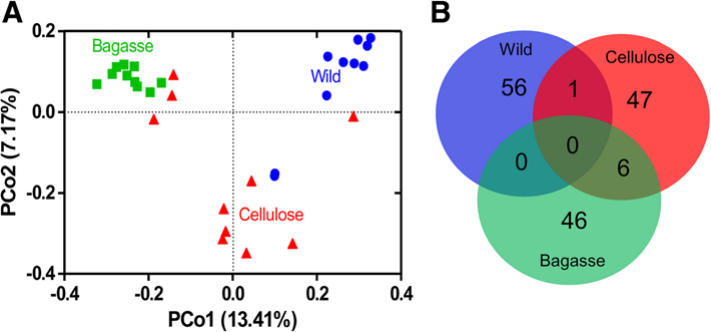
**Match between bacterial communities in cockroaches fed different diets. (A)** Similarity between bacterial communities. Principal coordinates plots (PCA) were generated using the pair wise unweighted UniFrac distances. □ Wild-types insects; Δ Insects fed sugarcane bagasse; ○ Insects fed cellulose; **(B)** Venn diagram with OTUs grouped at 97% similarity in the phylogenetic tree of bacterial clones obtained from cockroaches fed with different diets.

No OTUs were shared among the three groups analyzed in this work (Figure 4). Venn diagram shows that only one OUT from the wild-types insect library was shared with the cellulose fed group while none was shared between wild-types and sugarcane fed insects (Figure 4B). Six OTUs were shared between insects fed sugarcane bagasse and cellulose (mainly bacilli). Libraries were randomly sub-sampled and then community similarity was quantified based on phylogenetic relatedness by unweight UniFrac in a PCA plot (Figure 4A). In the scatter plot, the first two principal coordinates, PC1 and PC2, explained 13.4% and 7.1% of the data variation, respectively, clearly separating each community. These results suggest that there is a possible specialization of intestinal microbiota associated with a fiber-rich diet in both groups.

**Figure 5:**
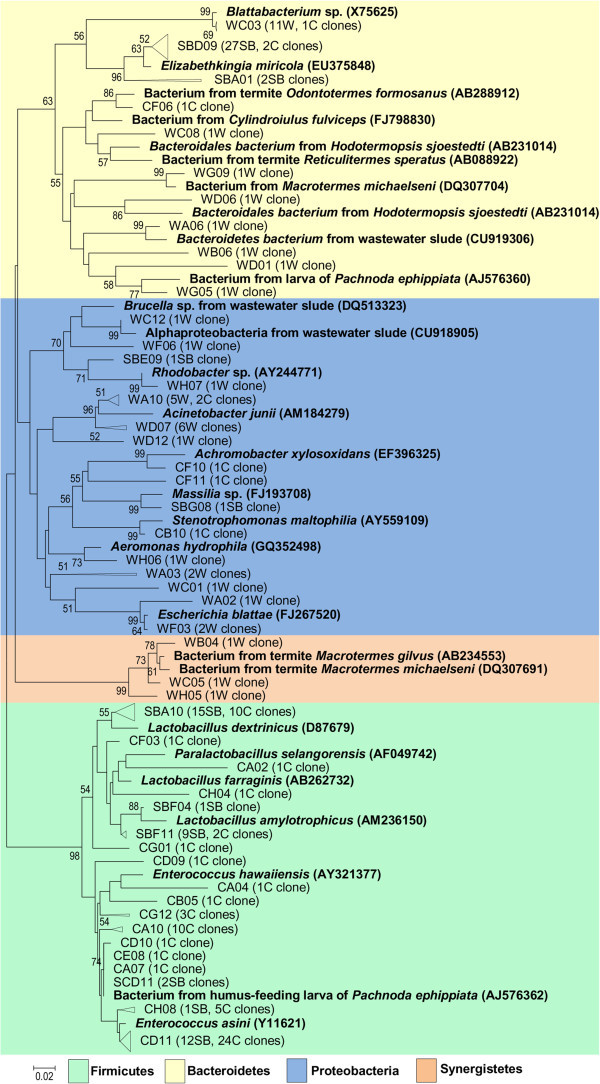
**Neighbor-joining phylogenetic tree of 16S rRNA gene sequences.** Cockroach bacterial clones from SB (fed on sugarcane bagasse), C (fed on cellulose) or W (wild-types). Reference sequences were retrieved from Genbank (in bold). Phylogenetic trees were constructed for *P. americana* gut 16S rRNA bacterial sequences with reference sequences from GenBank by the neighbor-joining algorithm based on distances calculated by the Kimura-2 method.

The phylogenetic tree showing the bacterial phylotypes retrieved from our clone libraries are shown in Figure 5. The wild-types cockroach group (W) shows OTUs mainly related to three groups: *Proteobacteria*, *Bacteroidetes* and *Synergistetes*. Clones corresponding to *Proteobacteria* are assigned to several different genera such as *Brucella, Rhodobacter, Acinetobacter, Aeromonas* and *Escherichia.* Clones representing the *Bacteroidetes* are mainly distributed through several genera such as *Blattabacterium, Elizabethkingia* and *Bacteroidales* while clones from *Synergistetes* are represented by three clones whose sequences are very similar to bacteria retrieved from the termite *Macrotermes gilvus*.

Among sugarcane bagasse (SB) fed insects, the *Firmicutes* and *Bacteroidetes* were the main phyla observed. *Firmicutes* are predominantly represented by the two genera *Lactobacillus* and *Enterococcus. Bacteroidetes* are exclusively represented by the genus *Elizabethkingia*. Among the *Proteobacteria*, few clones are assigned to the genera *Rhodobacter* and *Massilia*. Regarding cellulose (C) fed animals, the predominant sequences retrieved phyla were also *Firmicutes* and *Bacteroidetes*. In this group the *Firmicutes* are largely represented by the genera *Lactobacillus*, *Paralactobacillus and Enterococcus*. Five clones are similar to a bacterium from humus-feeding larva of *Pachnoda ephippiata.* The *Bacteroidetes* are principally represented by two genera, *Blattabacterium* and *Elizabethkingia*. Among the *Proteobacteria,* few clones are assigned to the genera *Acinetobacter, Achromobacter* and *Stenotrophomonas*.

## Discussion

This work studied the influence of diet on gut bacterial communities of *P. americana*. Here the 16S rDNAs sequencing approach was used to explore the plasticity of the intestinal flora of *Periplaneta americana*, comparing wild-types insects with others fed on different diets. The analysis of 16S rRNA sequences showed that the diet had a significant influence on gut microbial communities in this insect. It is important to note that the conditioning of these insects to different diets had no effect on animal survival rate during the study period (data not shown). Our results show clearly that there is a significant increase in *Firmicutes* presence and also an important decrease in Proteobacteria and Synergistetes in the gut of animals fed cellulose-rich diet (Figure 2).

An association between bacteria and insects during biomass degradation is observed in several models. The leaf-cutter ant *Atta colombicai*, for example, uses fresh leaves to cultivate a fungus and also bacteria (mainly γ-*Proteobacteria* of the family *Enterobacteriaceae*) that produce high numbers of different cellulases and hemicellulases in specialized biodegrading gardens (Suen et al. 2010). In higher termites (that do not have symbiotic protists in the gut), such as *Cornitermes cumulans* and *Nasutitermes* sp, a high bacterial diversity is observed, basically composed by the phyla *Proteobacteria, Spirochaeta, Bacteroidetes*, *Firmicutes*, *Actinobacteria*, *Fibrobacter* and *Treponema* (Gijzen et al. 1994; Grieco et al. 2012). Phylogenetic analysis of gut bacteria from the low termite *Reticulitermes flavipes* (that harbors protist symbionts in the gut) and *Cryptocercus* (wood-feeding cockroach) showed a diverse range of members of major bacterial phyla, such as *Proteobacteria*, *Spirochaetes*, *Bacteroidetes*, *Firmicutes*, *Actinobacteria, Synergistetes* and the newly proposed *Endomicrobia* (Fisher et al. 2007; Berlanga et al. 2009). The gut microbiota of the cockroach *Shelfordella lateralis* was dominated by members of the *Bacteroidetes* and *Firmicutes* (mainly *Clostridia*), however, *Deltaproteobacteria*, *Spirochaetes* and *Fibrobacteres*, which are abundant members of termite gut communities, were absent in this insect (Schauer et al. 2012).

The impact of diet on intestinal microbiota is also observed in other models such as dogs (Middelbos et al. 2010), snails (Cardoso et al. 2012), cattle (Kong et al. 2010; Hess et al. 2011), sheep (Cunha et al. 2011) and humans (Ley et al. 2006) for example. In the higher termite *Nasutitermes takasagoensis*, it was demonstrated that the intestinal bacterial community structure is not so stable, varying depending on diet composition. The *Spirochaetes* was predominant sequences in the wood-feeding termites, whereas *Bacteroidetes* was more abundant in the gut of xylophagous termites. *Firmicutes* was predominant sequences in xylose fed termites (Miyata et al. 2007). The analysis of the termite *Reticulitermes flavipes* gut microbiome submitted to different diets showed that diet, environment and host genetics have important effects over microbiome composition (Boucias et al. 2013). Our results showed that the main *Proteobacteria* clones associated to wild-types cockroaches are distributed along several genera, including typical bacteria from sludge such as *Brucella* and *Alphaproteobacteria*.

The presence of microorganisms in the gut of American cockroach *P. americana* was initially described by light, scanning and transmission electron microscopy. The results suggested the presence of a complex community including protozoa, bacteria and archaea (Gijzen and Barugahare 1992; Bignell et al. 1977). Previous studies showed that a cellulose rich diet induces an increase in the population of protozoa and also in methanogenesis in the hindgut of this insect 19-(Gijzen et al. 1994; Kane and Breznak 1991). In cockroaches and termites, the protists involved in lignocellulose degradation use not only their own enzymes, but also could use the enzymes originated from its endo and/or ecto symbiotic bacteria (Todaka et al. 2010). The greater complexity of nutrients within the wild-types and sugarcane bagasse diets compared to the simple diet (only cellulose) suggests that a greater repertoire of bacteria may be required to efficiently utilize all of the nutritional components of more complex foods. Results from our laboratory show that these bacterial community alterations associated with changes in dietary composition triggered some changes in the intestinal enzyme profile. For example, there is an important increase on Endo-1,3(4)-β-glucanase and Endo-beta-1,3-1,4 glucanase II activities of anterior intestine of insect fed sugarcane bagasse compared to other two groups (data not shown). An interesting hypothesis is that these specialized bacteria could help to digest complex dietary polymers during the passage through the first segments of the insect’s intestinal tract. After that, host enzymes could digest these bacteria as a nutrient source in the last gut segments as observed in flies with a strong participation of cathepsin-D-like proteases and lysozyme (Lemos and Terra 1991).

The exact role of the cockroach microbiota in biomass degradation still remains unknown. Bacteria from the phylum *Bacteroidetes* (formerly known as *Cytophaga-Flavobacteria-Bacteroides*-CFB group) are involved in associations with a wide variety of gut protist species as either intracellular endosymbionts or surface-attached ectosymbionts. These bacteria digest a wide variety of substrates, including complex polymers, such as cellulose and chitin, using various glycosyl hydrolases (Noda et al. 2006; Mahowald et al. 2009). It is important to note that all *Bacteroidetes* related clones retrieved from sugarcane bagasse fed insects are affiliated with *Elizabethkingia miricola*. These bacteria are Gram-negative, non-motile, that can utilize several glycosidic substrates such as D-fructose, D-glucose, D-maltose, 2-naphthyl-alpha-D-glucopyranoside, 1-naphthyl-N-acetyl-beta-D-glucosaminide and 2-naphthyl-alpha-L-fucopyranoside (Kim et al. 2005).

In the intestine (including in humans), *Firmicutes* are strongly involved in fermentation processes and may be partners in many catabolic activities such as those observed in the degradation of glucose to generate several catabolites as lactate, ethanol, H_2_ and CO_2_ (Ley et al. 2006; Wüst et al. 2011). These bacteria could also reduce sulfate, degrade volatile fatty acids, such as butyrate and its analogs, and provide H_2_ to archeal methanogens (Ley et al. 2008; Rivière et al. 2009). Most clones from cockroaches fed on a cellulose-rich diet (Sugarcane Bagasse and Cellulose groups) are distributed in two main distantly related species, *Lactobacillus dextrinicus* and *Enterococcus asini* that are examples of typical fecal bacteria (Furet et al. 2009). The *Synergistetes* phyla includes Gram-negative, rod-shaped bacteria isolated from humans, animals and terrestrial and oceanic bacteria that metabolize amino acids and proteins to provide short-chain fatty acids and sulfate for methanogens and sulfate-reducing bacteria (Vartoukian et al. 2007). Our data do not show the presence of bacteria from the genus *Clostridium*, which is an important group in the rumen of cattle involved in fiber degradation by the enzymatic complex called cellulosome (Ley et al. 2006). This result is also different from *Nasutitermes takasagoensis* where there is an important presence of *Clostridia* on the mixed segments of this higher termite (Tokuda et al. 2000).

The enzymatic repertoire involved in digestion of lignocelullosis in insects could include glycoside hydrolases, laccase, peroxidases and detoxification proteins such as superoxide dismutase and catalase (Scharf and Boucias 2010). Considering that insects like cockroaches and termites perform the pretreatment and hydrolysis under mild conditions within a few millimeters of intestinal tissue, our data demonstrate that there is an important specialization of the microbiota in fiber digestion. Thus, the knowledge about gut microorganisms and their enzymes involved in the pretreatment and hydrolysis of biomass could be useful for new insights related to the development of bioethanol or other high-value products.
